# Detection of locally adapted genomic regions in wild rice (*Oryza rufipogon*) using environmental association analysis

**DOI:** 10.1093/g3journal/jkad194

**Published:** 2023-08-24

**Authors:** James A Bedford, Mark Carine, Mark A Chapman

**Affiliations:** Biological Sciences, University of Southampton, Southampton SO17 1BJ, UK; Life Sciences, The Natural History Museum, London SW7 5BD, UK; Life Sciences, The Natural History Museum, London SW7 5BD, UK; Biological Sciences, University of Southampton, Southampton SO17 1BJ, UK

**Keywords:** local adaptation, *Oryza rufipogon*, environmental association analysis, climate, abiotic stress, genomics, domestication, wild rice, Plant Genetics and Genomics

## Abstract

*Oryza rufipogon* is the wild progenitor of cultivated rice *Oryza sativa* and exhibits high levels of genetic diversity across its distribution, making it a useful resource for the identification of abiotic stress–tolerant varieties and genes that could limit future climate-changed–induced yield losses. To investigate local adaptation in *O. rufipogon*, we analyzed single nucleotide polymorphism (SNP) data from a panel of 286 samples located across a diverse range of climates. Environmental association analysis (EAA), a genome-wide association study (GWAS)-based method, was used and revealed 15 regions of the genome significantly associated with various climate factors. Genes within these environmentally associated regions have putative functions in abiotic stress response, phytohormone signaling, and the control of flowering time. This provides an insight into potential local adaptation in *O. rufipogon* and reveals possible locally adaptive genes that may provide opportunities for breeding novel rice varieties with climate change–resilient phenotypes.

## Introduction

Domestication and selective breeding are necessary to produce high crop yields; however, crops tend to have a restricted genetic diversity when compared to their wild relatives (Jovovic *et al*. 2020) due to large genetic bottlenecks and selection (Flint-Garcia 2013). The reduction in diversity in domesticates is concerning for future food security as population genetic diversity is generally regarded as beneficial for adaptation to changing environments (Jump *et al*. 2009).

Rice (*Oryza sativa* L.) provides food for approximately 3.5 billion people, accounting for an estimated 50% of the caloric intake of half a billion people living in poverty in Asia (Muthayya *et al*. 2014). Globally, approximately 750 million tonnes of rice are produced per year, comparable with other cereal crops such as wheat (FAOSTAT 2023). The high yields of cultivated rice are a result of selective breeding, and *Oryza rufipogon* Griff., the wild progenitor of *O. sativa*, has greater levels of sequence diversity than both subspecies of *O. sativa* (subspecies *japonica* and *indica*; Caicedo *et al*. 2007; Huang *et al*. 2012). Analysis of rice yields in recent history (1979–2008) revealed that fluctuations in temperature and precipitation have had a considerable impact on yield (Ray *et al*. 2015) and climate modeling indicates that a 3°C increase in global mean temperature could reduce rice yield by up to 19% (Tao *et al*. 2011).

*O. rufipogon* has a perennial life cycle and occurs in permanent wetland environments, including ponds, marshes, rivers, and streams (Gao *et al*. 2012), preferring relatively deep water between 0.2 and 4 m (Vaughan 1994). An annual ecotype, referred to by some authors as *Oryza nivara* S.D. Sharma & Shastry, is found in shallow water and seasonally wet environments (Chang 1976; Li *et al*. 2006) and is recognized in this study as an *O. rufipogon* ecotype. Outcrossing rates of annual *O. rufipogon* range from 4 to 25.5% (Phan *et al*. 2012), whereas in perennial forms, this can reach 56% (Barbier 1989). The native range of *O. rufipogon* in its broad sense extends across East Asia, Indonesia, and northern Australia, located between latitudes of approximately 20° S and 30° N (Vaughan 1994), although the range is hypothesized to have shifted throughout its evolutionary history, coinciding with changes in climate patterns during the Holocene period (Dodson *et al*. 2021).

*O. rufipogon* is distributed widely across a range of ecological environments, potentially generating selection pressures resulting in locally adaptive variants in the genome, that is, genetic variants linked to increased fitness of populations in their native environment when compared to other introduced populations. This could stem from resistance to pests and pathogens, or the abiotic environment, for example seasons, temperature, and precipitation. In cultivated rice, crown root density is associated with high fitness under drought conditions (Groen *et al*. 2022) and flowering time varies across latitude, with locally adapted loss of function variants detected in key flowering time genes (reviewed by Shrestha *et al*. 2014). Investigations into the genetics of local adaptation in wild rice are limited, but there may be some overlap of genes involved in local adaptation with those in cultivated rice, although this has not been investigated.

Climate modeling and archaeobotanical information have revealed that both domesticated rice and wild rice have been influenced by shifts in global climate patterns. Spatial and temporal niche modeling of the *japonica* subspecies and integration with archaeological data suggest that declines in temperature played a role in shaping its distribution with minor changes in temperature predicted to have caused a large decline of tropical *japonica* in Northeast China (d’Alpoim Guedes *et al*. 2015). It is also suggested that these changes in temperature generated adaptive pressure resulting in the formation of temperate *japonica* from the tropical subspecies (d’Alpoim Guedes *et al*. 2015), which is supported by modeling and archaeological rice records (Gutaker *et al*. 2020). Overall, previous studies have thus highlighted the significant impact of climate on rice evolution and distribution and have identified traits linked to local adaptation in the domesticated *O. sativa* and its wild relative *O. rufipogon*.

High genetic diversity is a main characteristic of crop wild relatives (Zhang *et al*. 2017) and makes *O. rufipogon* a suitable model for the identification of abiotic stress–tolerant varieties and genes, with implications for the improvement of rice and other crops. There are multiple approaches to investigate the genetics of local adaptation in plant species, for example quantitative trait loci (QTL) mapping (Savolainen *et al*. 2013) or analysis of genetic markers though environmental association analysis (EAA) (Hoban *et al*. 2016). EAA uses statistical models to associate environmental variation with genetic variation, typically single nucleotide polymorphisms (SNPs), across hundreds of accessions or populations of a species (Rellstab *et al*. 2015). Genes for environmental adaptation will be located in, or near to, associated regions. These locally adaptive genes may be offset from significantly associated SNPs, as a result of linkage disequilibrium (LD), and require further analysis to be confident of the adaptive value.

Applications of EAA to investigate local adaptation and identify genetic variation associated with environmental conditions have been demonstrated in a few crop wild relatives, for example the wild progenitors of barley [*Hordeum vulgare* subsp. *spontaneum* (K. Koch) Thell; Lei *et al*. 2019] and soybean (*Glycine soja* Siebold & Zucc; Anderson *et al*. 2016). Therefore, EAA can be used for identification of genetic markers for crop breeding or identification of genes with potential functions in environmental response.

In this study, we investigate local adaptation in *O. rufipogon* with a view to investigating the genetic basis of local adaptations associated with aspects of the abiotic environment, with potential for rice breeding for future or more varied climates. We analyzed genome-wide SNP data from a subset of the wild *O. rufipogon* accessions sequenced by Huang *et al*. (2012), using EAA to identify environmentally associated genomic regions. Genes within these regions were collated and their functions analyzed in the context of previous literature, with the aim of identifying consistencies or novel differences between domesticated rice and its wild relative. The study provides an insight into potential local adaptation in wild rice and highlights several genes that may be involved in adaptation to environmental conditions.

## Methods

### Diversity panel

The diversity panel of wild rice used in this investigation is derived from a set of 446 *O. rufipogon* sensu lato accessions (i.e. including samples referable to *O. nivara*) located in the OryzaGenome database release 1.0 (Ohyanagi *et al*. 2016) and originally sequenced by Huang *et al*. (2012). These sequencing data are a collection of low-coverage (average 1.9×) whole-genome sequence data, for which SNPs were previously called by alignment to the *O. sativa* reference genome (IRGSP 4.0) with Smalt (v 0.4), keeping uniquely mapping reads with >96% identity, and identified by the Ssaha Pileup package (v 0.5), removing low-quality bases. Missing genotypes were imputed using a k-nearest neighbor imputation method with a reported accuracy of approximately 98% (Huang *et al*. 2010, 2012).

### Climate data

Initial checks of the 446 accessions resulted in the removal of 18 accessions without location data and correcting the country of origin for an additional 11 samples. Climate data were obtained from the WorldClim database version 1.4 (Hijmans *et al*. 2005) using the R package Raster (Hijmans *et al*. 2020) in RStudio (R Core Team 2022). The WorldClim data set contains global climate information interpolated from observed data collected between approximately 1960 and 1990 and organized into ca. 1-km^2^ grids (30 arc-second resolution grid, 0.86 km^2^ at the equator). Nineteen bioclimatic variables (9 temperature-associated, 6 precipitation-associated variables, and 4 associated with both temperature and precipitation; Supplementary Table 1) were retrieved from the data set using the accession location coordinates from Huang *et al*. (2012) to download specific grid values. Elevation values for each sample were obtained using the Raster “getData” function and the SRTM 90-m resolution data set. Three accessions were removed as elevation data could not be extracted for these locations.

Rellstab *et al*. (2015) recommended that populations showing extreme environmental values should not be included in analyses to reduce false positives arising through chance association with outliers. Consequently, rice accessions that were sampled from extreme climates (i.e. at least 1 variable was outside the 1.5× interquartile range) were identified and removed. This reduced the number of accessions to 286.

Composite climate variables, principal component 1 (PC1) and PC2, were derived from the climatic data via principal component analysis (PCA) on all climate variables using the RStudio core stats function “princomp” with parameters cor and scores enabled. The first and second principal component values were then extracted. Correlations between variables at each sample location were analyzed with Spearman's rank correlation and visualized using the R package “ggcorrplot” (Kassambara 2019). Based on the correlation data, groups of highly correlated variables (*ρ* ≤ −0.8; *ρ* ≥ 0.8) were reduced to a single variable to limiting the effects of multiple testing. Therefore, 13 environmental variables were analyzed here.

### *O. rufipogon* accessions and processing of genetic data

SNP data from the 286 accessions were processed in RStudio version 3.6.0. Missing data were recoded from “-” to “N”, and the data set was converted to “HapMap” style. SNPs were allocated IDs, e.g. ORRUF01_00001445T:A, detailing the species (ORRUF), chromosome (01), the location within the chromosome (00001445), and the reference/SNP base present (T:A).

On average, accessions were missing 6.78% of SNPs after imputation, with a maximum of 13% missing data; therefore, no further accessions were removed from the analysis because of missing data, resulting in a SNP count of 2,463,549. The proportion of missing data for each SNP ranged from 0 to 77.3%, and SNPs with greater than 20% missing data were removed, in line with previous studies (e.g. Lei *et al*. 2019), resulting in 2,267,618 SNPs. Finally, SNP minor allele frequency (MAF) was calculated and SNPs with a MAF of ≤0.05 were excluded, resulting in 1,898,737 SNPs for the EAA.

### EAA

The genome-wide association study (GWAS) program GAPIT3 (Lipka *et al*. 2012) was selected for the EAA using the single locus mixed-linear model (MLM). This approach has been used in other similar EAA studies (e.g. De La Torre *et al*. 2019; Lei *et al*. 2019). Typically, GAPIT identifies associations between phenotype and genotype data collected from a genetically and phenotypically diverse accession panel. This is achieved using MLMs that account for underlying population structure using PCA of SNP data, in our case, the first 3 principal components, and incorporating estimates of relatedness between accessions by producing a VanRaden kinship matrix. However, in EAA using GWAS programs, phenotype data are substituted with environmental data, adding the assumption that local adaptation is occurring in the studied population. Significant SNPs were corrected for multiple testing with false discovery rate (FDR)-adjusted *P* values and use of the Bonferroni multiple test correction threshold. Manhattan plots (Supplementary Fig. 1) and quantile–quantile (QQ) plots (Supplementary Fig. 2) were produced to visualize the model outputs.

### EAA SNP regions, gene identification, and analysis

SNPs significantly associated with the climate variables are often clustered into regions due to LD, including putatively neutral SNPs that are in close proximity to a selectively advantageous SNP variant. Regions were identified based on the 0.05 FDR-corrected *P* value and Bonferroni significance thresholds. Additional regions were identified from single SNPs if they were highly significant, passing the Bonferroni threshold. For gene identification, regions were extended by 10 kb to capture those that may be linked to the region by LD. Genes within regions were determined by comparing the chromosomal locations to the IRGSP Ver1.0 genome browser with MSU Osa1 gene model tracks (http://viewer.shigen.info/oryzagenome2/mapview/MapView.do).

Gene ontology (GO) term enrichment analysis was carried out on the genes present in the genomic regions identified from the EAA using AgriGO V2.0 (Tian *et al*. 2017), selecting singular enrichment analysis (SEA), an FDR significance threshold of 0.05 and using the MSU 7.0 gene ID reference consisting of 24,075 genes with annotated “complete GO” GO terms. Two sets of genes were analyzed, those associated with at least 1 temperature variable (BIO1, 4, 5, and 8 and PC1) and those associated with at least 1 precipitation variable (BIO8, 13, and 14 and PC1). Some overlap occurs between these categories due to a subset of the environmental variables being linked to both temperature and precipitation metrics. The temperature category input 59 genes, with 28 being annotated with GO terms, and the precipitation group inputting 52 genes with 24 annotated. Genic SNPs were also identified through alignment to the MSU Osa1 gene model and nonsynonymous SNPs assigned a Grantham score to determine the impact of amino acid sequence changes (Grantham 1974).

## Results

### *O. rufipogon* is distributed across a diverse range of environments

**Fig. 1.**
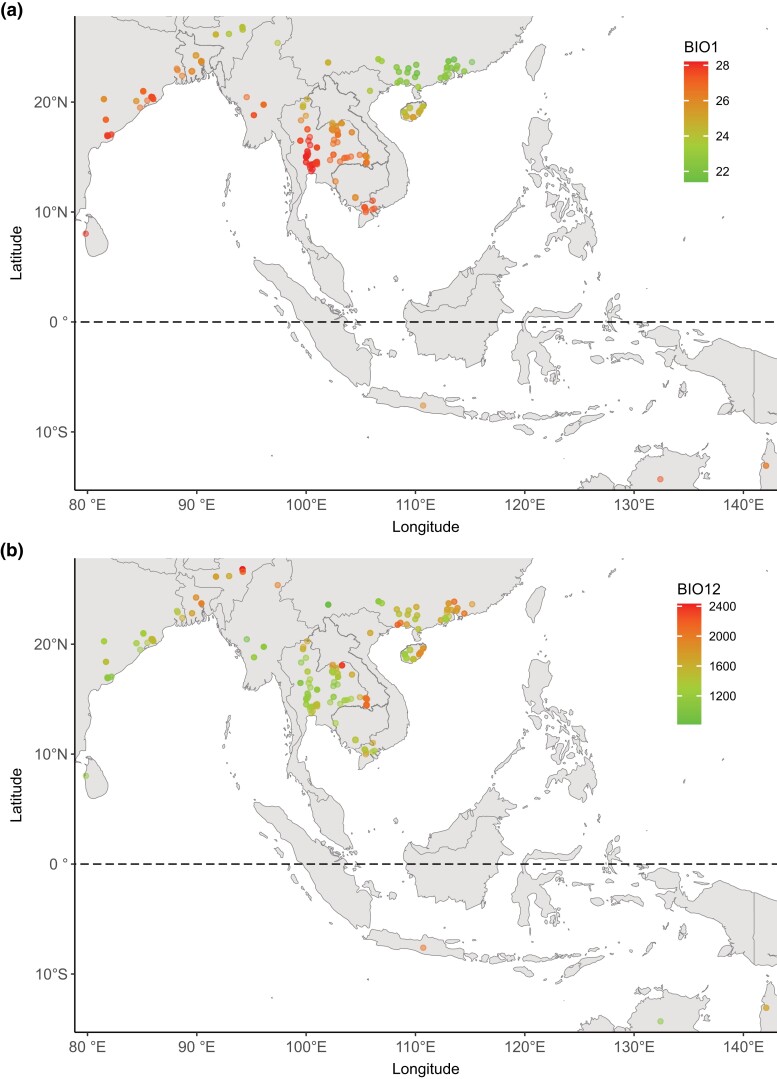
a) Mean annual temperature (°C; BIO1) and b) mean annual precipitation (mm; BIO12) across the 286 *O. rufipogon* sample locations.

The climate variables comprise 19 bioclimatic variables and elevation. Across the sample locations, average annual temperature (BIO1) ranges from 21.4°C to 28.1°C, generally decreasing with latitude (Fig. 1a). Annual precipitation (BIO12) displays a more heterogenous pattern than the temperature variables and varies 3-fold across the sample range, between ca. 820 and 2,430 mm (Fig. 1b). There is a high degree of correlation between the climatic variables, latitude and longitude, with strong correlations between most temperature-associated variables and between precipitation variables (Supplementary Fig. 3). Based on this correlation data, groups of highly correlated variables (*ρ* ≤ −0.8; *ρ* ≥ 0.8) were reduced to a single variable for the EAA, resulting in analysis of 13 variables.

**Fig. 2.**
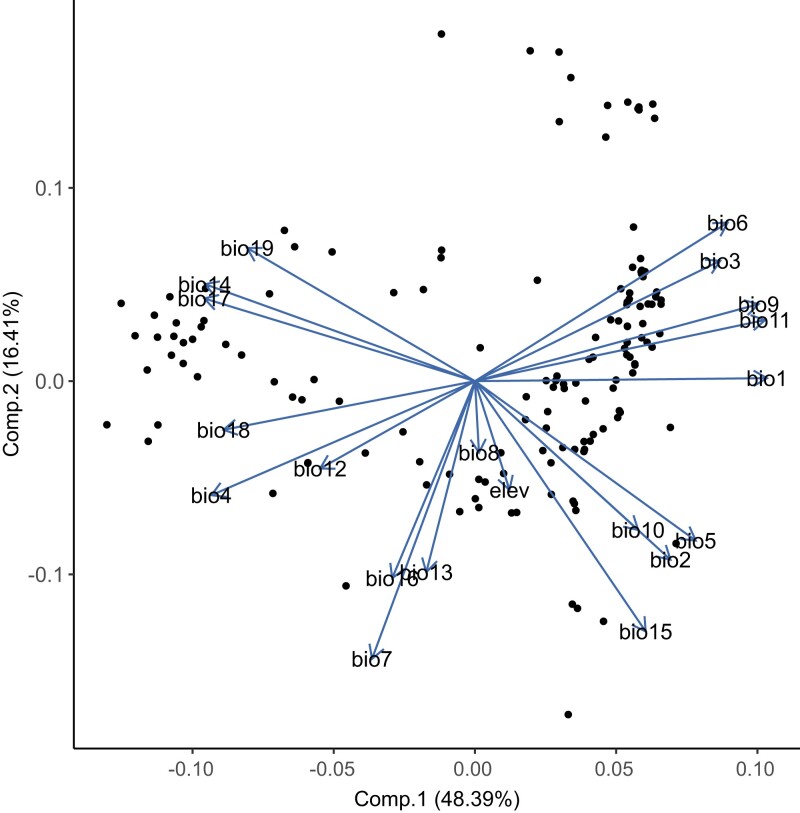
Accession distribution based on primary and secondary principal components derived from the environmental data. Points correspond to individual rice accessions, and arrows are the variable loadings, annotated with the variable name. Percentages represent the amount of variation explained by each axis.

Composite variables were derived from the climate data using PCA, and the first and second PCs were used in the EAA. The greatest loadings for PC1 include BIO1, 4, 9, 11, 14, and 17 (Fig. 2; Supplementary Table 2), which are measurements of temperature or precipitation in dry periods. Loadings for PC2 include BIO3, 6, 15, and 19, which are a variety of measurements related to isothermality and seasonality. In the environmental PCA (Fig. 2), samples with low PC1/intermediate PC2 values are predominantly from China, with a cooler climate, large annual temperature variation, and consistently higher precipitation. Samples with high PC1/intermediate PC2 values are from hot climates with average annual temperature range and maximum temperature and low precipitation. The samples with the greatest PC2 have consistently warm temperatures throughout the year, and samples with low PC2 values are from environments with high annual temperature variation and the highest maximum temperatures.

Previous population structure analyses on the SNP data set using neighbor-joining method revealed 3 genetic groups, Or-I, II, and III (Huang *et al*. 2012). These show patterns of geographic distribution, with Or-III predominantly found in China and West India, and Or-I and II displaying a more mixed distribution. This is reproduced in a PCA of the SNP data, revealing that the samples originating from China are more genetically distant and that the 2 genetic groups display mixed origins (Supplementary Fig. 4).

### Identification of genomic regions linked to environmental variables

The EAA was run using GAPIT, based on 1,898,737 SNPs across 286 individuals. Overall, 8 of 13 variables produced significant associations (Supplementary Fig. 1). Variables that failed to produce significant associations in the EAA included PC2, elevation, annual range in temperature (BIO7), annual precipitation (BIO12), and precipitation seasonality (BIO15). PC1 produced associations similar to other highly correlated temperature variables, and no genomic regions were associated with PC2.

Regions (R) were named by chromosome and then sequentially if more than 1 region was present on a chromosome. In total, 15 regions across 9 of the 12 chromosomes were identified, with each passing at least the FDR threshold and most passing the more stringent Bonferroni threshold (Table 1). Two of these associations represent significant associations for 2 or more of the environmental variables. For example, environmentally associated region 2.1 (R2.1) was identified on chromosome 2 for variables PC1, BIO1, and BIO4 (Table 1), all of which are various measurements of temperature. Similarly, 4 variables were associated with region R5.1.

**Table 1.** Fifteen environmentally associated regions, each assigned a chromosome/region ID.

Chr/region ID	Associated trait	Region start position (kb)	Region end position (kb)	Region size (kb)	Region type	Gene count (in region)	Gene count (within 10 kb)	Lowest FDR-adjusted *P* value	Threshold passed
R1.1	BIO14	2,688	2,691	3	Region	1	5	2.8 × 10^−4^	Bonferroni
R1.2	BIO13	30,002	30,003	1	Single	0	5	1.2 × 10^−2^	Bonferroni
R1.3	BIO5	39,215	39,235	20	Region	1	5	8.4 × 10^−3^	FDR
R2.1	PC1; BIO1; BIO4	12,523	12,550	27	Region	1	2	1.1 × 10^−2^	Bonferroni*^a^*
R2.2	BIO8	21,547	21,548	1	Region	0	2	6.2 × 10^−3^	Bonferroni
R2.3	BIO2	21,901	21,902	1	Region	0	3	2.2 × 10^−3^	Bonferroni
R3.1	BIO8	28,175	28,176	1	Single	0	3	1.7 × 10^−2^	FDR
R5.1	PC1; BIO1; BIO4; BIO14	16,122	16,140	18	Region	2	3	2.8 × 10^−4^	Bonferroni*^b^*
R6.1	BIO13	7,257	7,258	1	Single	0	4	1.2 × 10^−2^	Bonferroni
R6.2	BIO8	24,219	24,220	1	Single	0	6	7.4 × 10^−3^	Bonferroni
R7.1	BIO5	9,098	9,247	147	Region	7	9	1.0 × 10^−3^	Bonferroni
R8.1	BIO13	22,882	22,883	1	Single	1	4	1.2 × 10^−2^	Bonferroni
R10.1	BIO14	16,192	16,195	3	Region	0	2	2.8 × 10^−4^	Bonferroni
R12.1	BIO14	4,400	4,425	25	Region	3	4	2.6 × 10^−3^	Bonferroni
R12.2	BIO14	26,256	26,257	1	Region	0	0	4.3 × 10^−3^	Bonferroni

Listed are the associated trait(s), the genomic position of region boundaries, whether the region is formed from a single SNP or forms a peak region of multiple SNPs, the number of genes found within each region or in/within 10 kbp of the region, excluding transposable elements, details on the most significant SNP in each region, and the most stringent threshold passed.

PC1 (Bonferroni); BIO1 (Bonferroni); BIO4 (FDR).

PC1 (Bonferroni); BIO1 (FDR); BIO4 (Bonferroni); and BIO14 (Bonferroni).

The size of each significant region varied considerably, from a single significantly associated SNP to large regions of over 100 kb containing several significant SNPs after FDR correction. This could be a result of differences in sequence coverage or imputation in these regions, degree of LD, or the presence of multiple genes that are associated with the environmental variables.

### Identification of genes linked to environmental variables

Overall, 86 genes were present within or close to (within 10 kb) the 15 genomic regions (Supplementary Table 3). Several of these genes demonstrate putative functions that could be involved in local adaptation. Of the genes located in the environmentally associated regions, 7 contain an FDR-corrected SNP within their coding sequence, and 3 of these contain 1 or more nonsynonymous SNP (LOC_Os01g05640, LOC_Os01g67500, and LOC_Os05g27670; Table 2). Grantham scores indicate that 2 SNPs present in a receptor-like protein kinase (LOC_Os01g05640) will have low impact on the structure, whereas the serine to cysteine substitution for the armadillo/beta-catenin repeat family protein (LOC_Os01g67500) and premature stop codon in the uncharacterized protein (LOC_Os05g27670) will likely have a larger effect on the protein function. A GO enrichment analysis was also conducted, comparing the GO terms of genes located within regions associated with temperature or precipitation variables against a reference data set of GO terms. However, no significant enrichment was detected after correcting for multiple tests (Supplementary Table 4).

**Table 2.** Details about the FDR-significant SNPs located within predicted coding sequences of 7 genes and predicted sequence changes for the non-synonymous SNPs.

Gene model	Strand	Annotation	SNP	Amino acid change	Grantham score
LOC_Os01g05640	−	Receptor-like protein kinase 5 precursor	ORRUF01_02688980G:C		
			ORRUF01_02689466G:A		
			ORRUF01_02689630A:T	phe > ile	21
			ORRUF01_02690264T:C	lys > arg	26
			ORRUF01_02690615C:T		
			ORRUF01_02690861A:G		
			ORRUF01_02690885G:A		
LOC_Os01g67500	−	Armadillo/beta-catenin repeat family protein	ORRUF01_39223051G:C	ser > cys	112
			ORRUF01_39224857G:A		
LOC_Os02g35860	+	Expressed protein	ORRUF02_21547147T:C		
LOC_Os05g27670	+	Expressed protein	ORRUF05_16124784G:A	trp > stop	*^a^*
LOC_Os06g13215	+	Growth regulator–related protein	ORRUF06_07257355C:T		
LOC_Os07g15680	−	Phospholipase D	ORRUF07_09109350G:A		
LOC_Os08g36320	−	Glutamate decarboxylase	ORRUF08_22882132G:A		

Premature stop codon results in a predicted 11 amino acid truncation.

### Chromosome 1

Three environmentally associated regions were detected in chromosome 1. R1.1 was associated with the precipitation of the driest month, another (R1.2) shows association with precipitation of the wettest month, and the third (R1.3) is in association with the warmest month.

R1.1 contains a receptor-like protein kinase of unknown function, a disease resistance (R) gene *PYRICULARIA ORYZAE RESISTANCE T* (*PIT*; LOC_Os01g05630) and a metallothionein gene (*MT2a*; LOC_Os01g05650) ca. 2-kb downstream of this region. R1.2 is ca. 6 kb from a sulfate transporter (*SULTR3;6*; LOC_Os01g52130), which has previously been shown to be upregulated in drought and salinity stresses (Kumar *et al*. 2011). Additional genes within the region include those encoding hypothetically expressed proteins and a heavy metal–associated protein (HMP5; LOC_Os01g52160). Finally, R1.3 contains 3 genes with the annotations DNA-binding protein (LOC_Os01g67480), OTU-like cysteine protease (LOC_Os01g67490), armadillo/beta-catenin repeat family protein (LOC_Os01g67500), and a GDP-L-galactose phosphorylase (*GGP*; LOC_Os01g67520) is located downstream of this region and is predicted to function in the biosynthesis of ascorbic acid (Akram *et al*. 2017).

### Chromosome 2

Three environmentally associated regions were detected in chromosome 2. R2.1 is associated with several highly correlated temperature-associated variables. This region contains several retrotransposons and a gene encoding an F-box/Kelch repeat protein (*OsFBOX84*; LOC_Os02g21110), which is differentially expressed under light and dark conditions (Jain *et al*. 2007). The nonreference allele is predominantly in regions with low PC1 values, which corresponds to regions with lower temperatures.

The final 2 regions in chromosome 2 are associated with temperature of the wettest quarter and mean diurnal range, respectively. R2.2 contains an uncharacterized gene encoding an expressed protein (LOC_Os02g35860), and R2.3 has a terpene synthase gene, ent-isokaur-15-ene synthase (*OsKS6*; LOC_Os02g36264), which is located 1.9-kb upstream of the single significant SNP in this region.

### Chromosome 3

In chromosome 3, a single significant SNP peak was detected and associated with the mean temperature of the wettest quarter (BIO8). This SNP is near a gene encoding an ethylene receptor, *ETHYLENE RESPONSE SENSOR 1* (*OsERS1*; LOC_Os03g49500).

### Chromosome 5

R5.1 is associated with the precipitation of the driest month (BIO14), PC1, annual temperature, and temperature seasonality. This region contains a single gene encoding an expressed protein (LOC_Os05g27680) and a transposon protein.

### Chromosome 6

In chromosome 6, 2 significant regions were detected. R6.1, associated with the precipitation of the wettest month (BIO13), contains a predicted growth regulator, *O*-fucosyltransferase (LOC_Os06g13215), and R6.2, associated with the mean temperature of the wettest quarter (BIO8), is close to a predicted MADS-box transcription factor (LOC_Os06g40609) and an ESCRT-III complex SNF7 component (LOC_Os06g40620).

### Chromosome 7

**Fig. 3.**
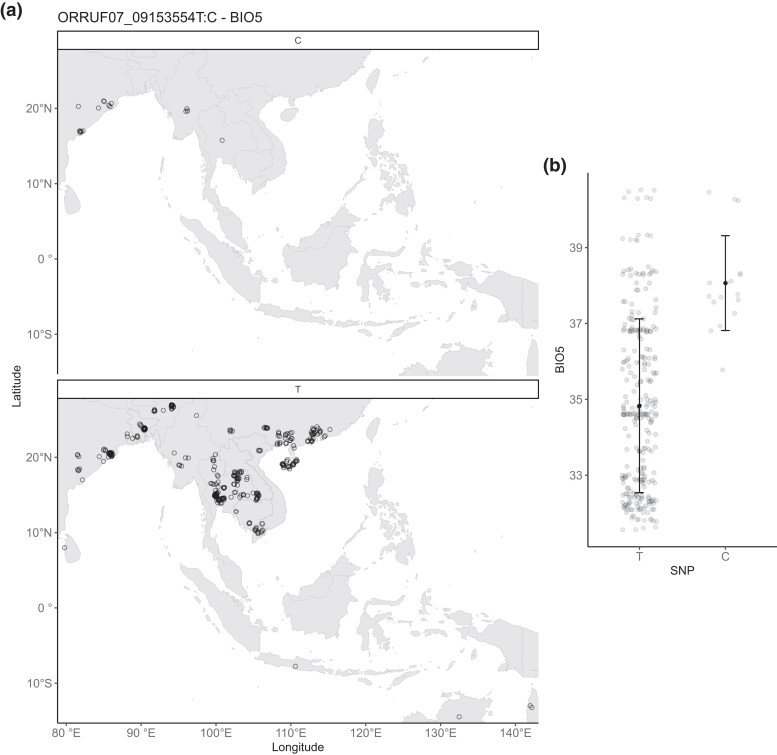
a) Distribution of SNPs across the diversity panel. Points represent the approximate locations of accessions (overlapping points offset for clarity), separated by the SNP ORRUF07_09153554T:C in the region R7.1, which is associated with BIO5 and contains 26 genes including the flowering time gene Ghd7. b) Association between the SNP and BIO5 maximum temperature of the warmest month (°C). Each point represents an individual accession, with mean ± 1 SD overlayed.

R7.1 was found to be associated with the maximum temperature of the warmest month (BIO5). This large 147-kb region contains 26 genes, 22 of which are genes of unknown function or transposons. Genes with functional annotations include genes putatively related to flowering time (*OsGHD7-like*; LOC_Os07g15770), reactive oxygen species (ROS) antioxidant activity (LOC_Os07g15670), and leaf and spikelet development (*NAL8*; LOC_Os07g15880). *OsGhd7* is linked to adaptation of rice to higher latitudes (Koo *et al*. 2013), and a SNP within *Ghd7* associated with BIO5 is found in accessions located in environments with high maximum temperatures (Fig. 3).

### Chromosome 8

R8.1 is associated with precipitation of the wettest month (BIO13) and contains a glutamate decarboxylase (*OsGAD1*; LOC_Os08g36320), with the SNP allele present in accessions found in climates with relatively low precipitation in their wettest month. *OsGAD1* has high sequence similarity to *AtGAD1* (AT5G17330: 94% coverage; 86.97% identity), which is implicated in the response to heat, drought, and hypoxia stresses, through production of GABA (Kinnersley and Turano 2000).

### Chromosome 10

Analysis of the precipitation of the driest month produced an additional SNP peak in chromosome 10 (R10.1) near genes encoding ubiquitin-conjugating enzyme 10 (*OsUBC10*; LOC_Os10g31000) and mRNA adenosine methylase 4 (*OsMTA4*; LOC_Os10g31030). Although the function of these proteins in rice is largely unknown, expression of *OsUBC10* is upregulated by the phytohormone abscisic acid (ABA) (E *et al*. 2015).

### Chromosome 12

In chromosome 12, there are 2 significant regions both associated with BIO14. R12.1 consists of 3 genes with unknown functions, and R12.2 does not contain any genes within 10 kb. The closest gene is 11.2-kb downstream and is predicted to encode a 9-*cis*-epoxycarotenoid dioxygenase (NCED2; LOC_Os12g42280).

Overall, a large proportion of the environmentally associated SNPs were near genes with unknown functions, highlighting the importance of gene annotation in model systems. Despite this, 8 of the 15 regions identified contain genes with putative functions linked to response to abiotic/biotic conditions or hormone signaling. This included genes known to be regulated by or implicated in ethylene and ABA signaling and the GABA shunt pathway. Genes functioning in response to temperature, drought, UV, and hypoxia were present in several of the significant regions. While there was no significant enrichment of GO terms, this may indicate that the various adaptations are controlled by a range of processes.

## Discussion

The aim of the analysis was to identify genetic markers associated with variation in environmental conditions across the native range of *O. rufipogon*. Genes predicted to be in close proximity to these markers were identified and their biological functions were analyzed bioinformatically. This has allowed us to detect genomic regions potentially involved in the local adaptation of *O. rufipogon* and to highlight genes and mechanisms predicted to contribute to response to environmental conditions.

The diversity panel used in the analysis extends across most of the species’ range, although with limited sampling in Indonesia and West India. The environmental data set was summarized with a PCA, revealing that variables contributing the most to the PC1 loadings include those relating to temperature. This is similar to a previous analysis of Chinese *O. rufipogon*, which found that the variables annual temperature, temperature seasonality, and mean annual radiation were the main factors contributing to variation in climate across the population (Zhou *et al*. 2013). While some of the genome regions identified were associated with a single variable, others were associated with multiple (e.g. R2.1 and R5.1), meaning the specific environmental factor underlying the association cannot be confirmed and, in many cases, multiple overlapping EAA regions are likely due to the correlation between variables. It should also be noted that the climate variables may also correlate with variables not analyzed in this investigation, such as soil factors, exposure, or salinity. Here, we focus on genes potentially underlying local adaptation to temperature and precipitation.

### Detection of the heading date gene *Ghd7*, known to be involved in local adaptation in domesticated rice

One of the candidate genes in a region associated with temperature variables encodes the heading date gene *Ghd7*. In *O. sativa*, *Ghd7* is a key component of the flowering time pathway (Shrestha *et al*. 2014). *S*equence differences in *Ghd7* are associated with adaptation to long-day conditions (Shrestha *et al*. 2014), and naturally occurring haplotypes of *OsGhd7* have been associated with variation in heading dates and latitudes (Xue *et al*. 2008; Koo *et al*. 2013; Cui *et al*. 2020). Cultivars from the most northern latitudes (beyond the northern limits of *O. rufipogon*) are often associated with non-functional *Ghd7* variants (Xue *et al*. 2008).

In our analysis, populations with the nonreference allele were from locations with high maximum temperatures. Because high temperature stress during reproductive stages causes sterility (at least in domesticated rice; Jagadish *et al*. 2007), natural variation in *Ghd7* could alter flowering time, functioning to escape high temperature stress. Lifecycle shifts for stress avoidance are known to occur in *Arabidopsis thaliana* in response to a broad range of abiotic and biotic factors (Kazan and Lyons 2016) and in the wild oat species *Avena barbata* Pott ex Link (Sherrard and Maherali 2006). *OsGhd7* is also responsive to other stress conditions (Weng *et al*. 2014; Du *et al*. 2018) and hence could be involved in adaptation to other conditions.

### Detection of phytohormone-associated genes

Some of the EAA candidate genes are associated with phytohormones, which are often involved in stress response and developmental processes, for example ABA (Shi and Yang 2014; Muhammad Aslam *et al*. 2022) and ethylene (Müller and Munné-Bosch 2015). *OsGhd7*, mentioned before, is repressed by ABA and jasmonic acid (Weng *et al*. 2014). The gene *ERS1* is a predicted negative regulator of ethylene signaling, influences root length in *O. sativa* (Ma *et al*. 2014) and is upregulated in salinity (Hossain *et al*. 2016).

Finally, the ubiquitin-conjugating enzyme UBC10 is associated with precipitation of the driest month and quarter. UBCs are involved in the ubiquitination of target proteins (Ciechanover 1994) and can function in a variety of processes including developmental processes, hormone signaling (Dreher and Callis 2007), flowering time (Xu *et al*. 2009), and abiotic stress response (Lyzenga and Stone 2012). In *O. sativa*, *UBC10* expression is increased in response to ABA (Zhiguo *et al*. 2015), which is usually associated with the regulation of developmental processes (Rodríguez-Gacio *et al*. 2009) and response to abiotic stresses (Seki *et al*. 2002). In rice, ABA has also been linked to drought escape pathways through regulation of flowering time genes (Du *et al*. 2018). Therefore, UBC10 may function in ABA signaling to regulate developmental or stress response processes.

### Detection of abiotic stress–related genes

Some of the EAA candidate genes have possible functions in response to various abiotic stresses. A previous drought study in *O. sativa* identified large transcriptional changes in root and shoot tissue between drought and control conditions (Groen *et al*. 2022). Four of these differentially expressed genes were identified in this EAA, *OsMT2a*, a cytochrome p450, an expressed protein and *GAD1*. In addition to differential expression under drought, 2 genes are induced under other stress conditions too; *OsMT2a* expression is induced by heat shock (Hsieh *et al*. 1996), and *SULTR3;6* is responsive to salinity stresses (Kumar *et al*. 2011). It is possible that these genes confer local adaptation in *O. rufipogon*.

*GAD1* encodes an enzyme in the GABA-shunt pathway and was detected in association with precipitation. The GABA-shunt pathway is branched from the TCA cycle and is thought to function to support the respiratory metabolic system, as well as during stress responses by promoting seedling growth (Lee *et al*. 2021) and though ROS scavenging (Bouché and Fromm 2004), a mechanism used to maintain ROS homeostasis (Gill and Tuteja 2010). *GAD1* in *O. sativa* is upregulated during heat (Cao *et al*. 2013; Liao *et al*. 2015), drought (Groen *et al*. 2022), and cold stresses (Zeng *et al*. 2022), supporting the association identified in the EAA.

Finally, *FBOX84*, predominantly associated with temperature variables in our EAA, is downregulated in white light compared to dark conditions, but not for other stresses (Jain *et al*. 2007). It is possible that these environments correlate with UV, exposure, or shading, although the specific function of this gene remains unknown.

### Evaluation of the EAA method

It should be noted that GWAS-based methods have reduced detection power when analyzing complex traits as these are likely controlled by multiple additive loci with weak effects. Although the number of accessions is reasonably high in this study, the SNP sequence data are limited by low coverage, which would have provided less detection power in certain regions. This would especially occur if these regions are characterized by higher recombination rates breaking up the association between SNP alleles and causative genes. Due to the low coverage, the SNP data do not include heterozygous sites, which will be present in a wild species such as this, and so missing possible associations. The proportion of heterozygous sites has previously been calculated to range between 0.1 and 5.4% for 5 *O. rufipogon* accessions with greater than 9× sequencing depth (Huang *et al*. 2012). Further, the resequencing data were mapped to an older congener reference genome than is available now (e.g. Xie *et al*. 2021; Que *et al*. 2022) and so there may be genes in *O. rufipogon* that are missing from our analysis, although this can occur even within species (Zhao *et al*. 2018). Therefore, in future investigations, greater sequencing depth and either a new reference genome or conspecific reference could be used. Despite these limitations, the *O. rufipogon* SNP data set has previously been successfully analyzed using GWAS for traits such as tiller angle (Huang *et al*. 2012). The removal of multiple accessions during filtering steps may also reduce detection of associations; however, it increases the likelihood that the significant SNPs detected here are true positive results, providing a more conservative approach of detecting SNP–environment associations. Optimization of EAA analyses more broadly in terms of outlier removal, both in terms of SNPs and individuals, should be prioritized to ensure these analyses achieve the optimal balance between removing false positives and reducing false negatives.

The EAA only detects associated genomic loci and is unable to detect transcriptional responses to the environment. Related to this, LD can result in the causative gene being distant from a significantly associated SNP, adding to the challenge of confidently selecting candidate genes. LD decays relatively quickly in rice, dropping to half at around 20 kb (Huang *et al*. 2012); therefore, we assume that the causative genetic changes are physically relatively close to the outlier SNPs identified. The association between *Ghd7*, a gene known to be involved in local adaptation, and maximum temperature in the EAA (see Fig. 3) provides confidence that at least a subset of our associated genes and regions are true positive associations.

The absence of significant GO terms may be explained by LD; only a single gene may be causative in each environmentally associated region, but our GO analysis necessarily included all genes in EAA regions. In addition, for a significant portion of the associated genes, the annotations were weak; hence, some adaptively important genes may have been uncovered but remain with unknown function. Regardless of this limitation, associated regions could be used in marker-assisted selective breeding.

Taking these caveats into account, the *O. rufipogon* EAA revealed 15 regions of the genome associated with environmental variables and are predicted to contain several genes with functions potentially associated with abiotic stress, flowering, developmental processes, and hormone signaling. The investigation looks at the association between environmental conditions and genetic variation and consequently focuses on the abiotic component of local adaptation. Therefore, additional genomic regions may be detected in association with biotic factors. Although requiring confirmatory analysis, these loci provide significant potential for adaptive variation in the wild progenitor of one of the world's most widely grown crops. Going forward, alleles at these genes have the potential for being incorporated into breeding programs to ensure rice adaptation for a future climate.

## Supplementary Material

jkad194_Supplementary_Data

## Data Availability

The full genome sequencing data from Huang *et al*. (2012) are available from OryzaGenome (http://viewer.shigen.info/oryzagenome/mapview/Top.do). The processed SNP and climate data from the subset of individuals we used in our analysis are available online (doi: 10.6084/m9.figshare.21916038.v1). Supplemental material available at G3 online.
