# Supplementary material for: Detection of locally adapted genomic regions in wild rice (Oryza rufipogon) using environmental association analysis
Source: G3 (Bethesda). 2023 Aug 24;13(10):jkad194. doi: 10.1093/g3journal/jkad194 (PMC10542315; doi:10.1093/g3journal/jkad194)
Supplement: jkad194_Supplementary_Data [file jkad194_supplementary_data.zip › G3-2023-404430R1_Supplemental_Material_Legends.docx]

Supplementary material

**File S1 -** Contains Figures S1 to S4

**Figure S1 -** SNPs associated with the 10 bioclimatic variables, principal components one and two, and elevation. Each point represents a SNP. The dashed line is the Bonferroni significance threshold and colored SNPs pass FDR.

**Figure S2 -** Observed versus expected SNP p-values for each environmental variable input into the GAPIT mixed linear model. The red line illustrates a 1:1 relationship.

**Figure S3 -** Correlations between environmental and location variables. The scale corresponds to the Spearman’s rank correlation coefficient rho values. T = temperature-associated variable; P = precipitation; B = both temperature and precipitation; O = other variables.

**Figure S4 -** Population structure of the 286 *O. rufipogon* accessions, resulting from a PCA of the SNP data. Each accession is colored by its country of origin.

**Table S1 -** Bioclimatic and elevation variable keys and descriptions.

**Table S2 -** Loading scores for each bioclimatic variable across the first 20 principal components

**Table S3 -** Details on the 86 genes identified in or within 10kb of a significant environmentally associated region. Region ID corresponds to the chromosome (R1.1 and R1.2 are both in chromosome 1). Region boundaries are to the nearest kilobase.

**Table S4 -** Enrichment scores for GO terms associated with genes found in or within 10 kb of temperature-associated genomic regions and precipitation-associated regions.
