# Supplementary material for: Detection of locally adapted genomic regions in wild rice (Oryza rufipogon) using environmental association analysis
Source: G3 (Bethesda). 2023 Aug 24;13(10):jkad194. doi: 10.1093/g3journal/jkad194 (PMC10542315; doi:10.1093/g3journal/jkad194)
Supplement: jkad194_Supplementary_Data [file jkad194_supplementary_data.zip › G3-2023-404430R1_Table_S1.docx]

| **Table S1 –** Bioclimatic and elevation variable keys and descriptions. | |
| --- | --- |
| **Climate variable** | **Description** |
| BIO1 | Annual mean temperature (°C) |
| BIO2 | Mean diurnal range (mean of monthly [max temperature – min temperature]; °C) |
| BIO3 | Isothermality ((BIO2/BIO7) *100; %) |
| BIO4 | Temperature seasonality (standard deviation *100; °C*10) |
| BIO5 | Max temperature of warmest month (°C) |
| BIO6 | Min temperature of coldest month (°C) |
| BIO7 | Temperature annual range (BIO5-BIO6; °C) |
| BIO8 | Mean temperature of wettest quarter (°C) |
| BIO9 | Mean temperature of driest quarter (°C) |
| BIO10 | Mean temperature of warmest quarter (°C) |
| BIO11 | Mean temperature of coldest quarter (°C) |
| BIO12 | Annual precipitation (mm) |
| BIO13 | Precipitation of wettest month (mm) |
| BIO14 | Precipitation of driest month (mm) |
| BIO15 | Precipitation seasonality (coefficient of variation; %) |
| BIO16 | Precipitation of wettest quarter (mm) |
| BIO17 | Precipitation of driest quarter (mm) |
| BIO18 | Precipitation of warmest quarter (mm) |
| BIO19 | Precipitation of coldest quarter (mm) |
| ELEV | Elevation (m) |
