# Supplementary material for: Detection of locally adapted genomic regions in wild rice (Oryza rufipogon) using environmental association analysis
Source: G3 (Bethesda). 2023 Aug 24;13(10):jkad194. doi: 10.1093/g3journal/jkad194 (PMC10542315; doi:10.1093/g3journal/jkad194)
Supplement: jkad194_Supplementary_Data [file jkad194_supplementary_data.zip › G3-2023-404430R1_Table_S3.docx]

| **Table S3 -** Details on the 86 genes identified in or within 10kb of a significant environmentally associated region. Region ID corresponds to the chromosome (R1.1 and R1.2 are both in chromosome 1). Region boundaries are to the nearest kilobase. | | | | | | | |
| --- | --- | --- | --- | --- | --- | --- | --- |
| **Region ID** | **Associated variable(s)** | **Region start (kbp)** | **Region end (kbp)** | **Gene locus ID** | **Gene start (bp)** | **Gene end (bp)** | **Annotation** |
| R1.1 | bio14 | 2688 | 2691 | LOC_Os01g05620 | 2682019 | 2684988 | NBS-LRR disease resistance protein, putative, expressed |
| R1.1 | bio14 | 2688 | 2691 | LOC_Os01g05630 | 2686729 | 2687700 | Core histone H2A/H2B/H3/H4 domain containing protein, putative, expressed |
| R1.1 | bio14 | 2688 | 2691 | LOC_Os01g05640 | 2688623 | 2692186 | Receptor-like protein kinase 5 precursor, putative, expressed |
| R1.1 | bio14 | 2688 | 2691 | LOC_Os01g05650 | 2693134 | 2695085 | Metallothionein, putative, expressed |
| R1.1 | bio14 | 2688 | 2691 | LOC_Os01g05660 | 2698928 | 2703491 | Expressed protein |
| R1.2 | bio13 | 30002 | 30003 | LOC_Os01g52130 | 29987911 | 29994141 | Sulfate transporter, putative, expressed |
| R1.2 | bio13 | 30002 | 30003 | LOC_Os01g52140 | 29995196 | 29999996 | Expressed protein |
| R1.2 | bio13 | 30002 | 30003 | LOC_Os01g52150 | 30000720 | 30001375 | Hypothetical protein |
| R1.2 | bio13 | 30002 | 30003 | LOC_Os01g52160 | 30003532 | 30005946 | Heavy metal-associated domain containing protein, expressed |
| R1.2 | bio13 | 30002 | 30003 | LOC_Os01g52170 | 30008281 | 30012094 | Expressed protein |
| R1.3 | bio5 | 39215 | 39235 | LOC_Os01g67480 | 39211307 | 39216637 | Helix-loop-helix DNA-binding domain containing protein, expressed |
| R1.3 | bio5 | 39215 | 39235 | LOC_Os01g67490 | 39216579 | 39219456 | OTU-like cysteine protease family protein, putative, expressed |
| R1.3 | bio5 | 39215 | 39235 | LOC_Os01g67500 | 39222268 | 39226573 | Armadillo/beta-catenin repeat family protein, putative, expressed |
| R1.3 | bio5 | 39215 | 39235 | LOC_Os01g67510 | 39238786 | 39243986 | recA protein, putative, expressed |
| R1.3 | bio5 | 39215 | 39235 | LOC_Os01g67520 | 39244417 | 39247128 | VTC2, putative, expressed |
| R2.1 | PCAComp1; BIO1; BIO4 | 12523 | 12550 | LOC_Os02g21100 | 12509468 | 12515074 | Retrotransposon protein, putative, Ty3-gypsy subclass, expressed |
| R2.1 | PCAComp1; BIO1; BIO4 | 12523 | 12550 | LOC_Os02g21110 | 12528758 | 12533303 | OsFBK6 - F-box domain and kelch repeat containing protein, expressed |
| R2.1 | PCAComp1; BIO1; BIO4 | 12523 | 12550 | LOC_Os02g21120 | 12543435 | 12544141 | Transposon protein, putative, unclassified |
| R2.1 | PCAComp1; BIO1; BIO4 | 12523 | 12550 | LOC_Os02g21130 | 12545020 | 12548081 | Retrotransposon protein, putative, unclassified, expressed |
| R2.1 | PCAComp1; BIO1; BIO4 | 12523 | 12550 | LOC_Os02g21140 | 12548728 | 12550308 | Retrotransposon protein, putative, unclassified, expressed |
| R2.1 | PCAComp1; BIO1; BIO4 | 12523 | 12550 | LOC_Os02g21150 | 12552655 | 12554964 | Retrotransposon protein, putative, unclassified, expressed |
| R2.1 | PCAComp1; BIO1; BIO4 | 12523 | 12550 | LOC_Os02g21160 | 12558786 | 12559034 | Hypothetical protein |
| R2.2 | bio8 | 21547 | 21548 | LOC_Os02g35860 | 21543610 | 21548942 | Expressed protein |
| R2.2 | bio8 | 21547 | 21548 | LOC_Os02g35870 | 21549817 | 21555020 | Galactosyltransferase, putative, expressed |
| R2.3 | bio2 | 21901 | 21902 | LOC_Os02g36264 | 21886310 | 21899230 | Terpene synthase, putative, expressed |
| R2.3 | bio2 | 21901 | 21902 | LOC_Os02g36280 | 21904354 | 21906350 | Cytochrome P450, putative, expressed |
| R2.3 | bio2 | 21901 | 21902 | LOC_Os02g36285 | 21911434 | 21912631 | Terpene synthase family, metal binding domain containing protein, expressed |
| R3.1 | bio8 | 28175 | 28176 | LOC_Os03g49490 | 28164811 | 28167450 | Expressed protein |
| R3.1 | bio8 | 28175 | 28176 | LOC_Os03g49500 | 28169807 | 28174250 | Ethylene receptor, putative, expressed |
| R3.1 | bio8 | 28175 | 28176 | LOC_Os03g49510 | 28180227 | 28186143 | Phosphatidylinositol-4-phosphate 5-kinase, putative, expressed |
| R5.1 | PCAComp1; BIO1; BIO4; BIO14 | 16122 | 16140 | LOC_Os05g27660 | 16107806 | 16112248 | Expressed protein |
| R5.1 | PCAComp1; BIO1; BIO4; BIO14 | 16122 | 16140 | LOC_Os05g27670 | 16124192 | 16124785 | Expressed protein |
| R5.1 | PCAComp1; BIO1; BIO4; BIO14 | 16122 | 16140 | LOC_Os05g27680 | 16127304 | 16128208 | Expressed protein |
| R5.1 | PCAComp1; BIO1; BIO4; BIO14 | 16122 | 16140 | LOC_Os05g27690 | 16128850 | 16132454 | Transposon protein, putative, CACTA, En/Spm sub-class, expressed |
| R5.1 | PCAComp1; BIO1; BIO4; BIO14 | 16122 | 16140 | LOC_Os05g27700 | 16134484 | 16136936 | Transposon protein, putative, CACTA, En/Spm sub-class, expressed |
| R5.1 | PCAComp1; BIO1; BIO4; BIO14 | 16122 | 16140 | LOC_Os05g27710 | 16138914 | 16140698 | Transposon protein, putative, CACTA, En/Spm sub-class |
| R5.1 | PCAComp1; BIO1; BIO4; BIO14 | 16122 | 16140 | LOC_Os05g27720 | 16141193 | 16145937 | Transposon protein, putative, CACTA, En/Spm sub-class, expressed |
| R6.1 | bio13 | 7257 | 7258 | LOC_Os06g13210 | 7247729 | 7251058 | Peptide transporter PTR2, putative, expressed |
| R6.1 | bio13 | 7257 | 7258 | LOC_Os06g13215 | 7256821 | 7259221 | Growth regulator related protein, putative, expressed |
| R6.1 | bio13 | 7257 | 7258 | LOC_Os06g13220 | 7260407 | 7264016 | Expressed protein |
| R6.1 | bio13 | 7257 | 7258 | LOC_Os06g13230 | 7267160 | 7269232 | Expressed protein |
| R6.2 | bio8 | 24219 | 24220 | LOC_Os06g40600 | 24206615 | 24212569 | Elongation factor, putative, expressed |
| R6.2 | bio8 | 24219 | 24220 | LOC_Os06g40609 | 24215332 | 24217187 | Expressed protein |
| R6.2 | bio8 | 24219 | 24220 | LOC_Os06g40620 | 24218361 | 24223375 | SNF7 domain containing protein, putative, expressed |
| R6.2 | bio8 | 24219 | 24220 | LOC_Os06g40630 | 24223752 | 24226794 | SFT2, putative, expressed |
| R6.2 | bio8 | 24219 | 24220 | LOC_Os06g40640 | 24227263 | 24229290 | Fructose-bisphospate aldolase isozyme, putative, expressed |
| R6.2 | bio8 | 24219 | 24220 | LOC_Os06g40650 | 24229308 | 24234795 | Copine-1, putative, expressed |
| R7.1 | bio5 | 9098 | 9247 | LOC_Os07g15660 | 9090751 | 9095233 | Retrotransposon protein, putative, LINE subclass |
| R7.1 | bio5 | 9098 | 9247 | LOC_Os07g15670 | 9099097 | 9102892 | Peroxiredoxin, putative, expressed |
| R7.1 | bio5 | 9098 | 9247 | LOC_Os07g15680 | 9105495 | 9112919 | Phospholipase D, putative, expressed |
| R7.1 | bio5 | 9098 | 9247 | LOC_Os07g15690 | 9117116 | 9119948 | Transposon protein, putative, CACTA, En/Spm sub-class, expressed |
| R7.1 | bio5 | 9098 | 9247 | LOC_Os07g15700 | 9124904 | 9125431 | Transposon protein, putative, unclassified |
| R7.1 | bio5 | 9098 | 9247 | LOC_Os07g15710 | 9127630 | 9131795 | Transposon protein, putative, unclassified, expressed |
| R7.1 | bio5 | 9098 | 9247 | LOC_Os07g15720 | 9136930 | 9137349 | Hypothetical protein |
| R7.1 | bio5 | 9098 | 9247 | LOC_Os07g15730 | 9137637 | 9137996 | Retrotransposon protein, putative, Ty3-gypsy subclass, expressed |
| R7.1 | bio5 | 9098 | 9247 | LOC_Os07g15740 | 9138268 | 9142486 | Retrotransposon protein, putative, Ty3-gypsy subclass, expressed |
| R7.1 | bio5 | 9098 | 9247 | LOC_Os07g15750 | 9142769 | 9143767 | Retrotransposon protein, putative, Ty3-gypsy subclass |
| R7.1 | bio5 | 9098 | 9247 | LOC_Os07g15760 | 9144163 | 9147358 | Retrotransposon protein, putative, Ty3-gypsy subclass, expressed |
| R7.1 | bio5 | 9098 | 9247 | LOC_Os07g15770 | 9152402 | 9155185 | CCT motif family protein, expressed |
| R7.1 | bio5 | 9098 | 9247 | LOC_Os07g15780 | 9160841 | 9164568 | Retrotransposon protein, putative, Ty1-copia subclass, expressed |
| R7.1 | bio5 | 9098 | 9247 | LOC_Os07g15790 | 9167486 | 9170528 | Transposon protein, putative, CACTA, En/Spm sub-class, expressed |
| R7.1 | bio5 | 9098 | 9247 | LOC_Os07g15800 | 9172150 | 9177181 | Transposon protein, putative, CACTA, En/Spm sub-class, expressed |
| R7.1 | bio5 | 9098 | 9247 | LOC_Os07g15820 | 9188894 | 9189261 | Expressed protein |
| R7.1 | bio5 | 9098 | 9247 | LOC_Os07g15830 | 9194168 | 9196511 | Retrotransposon protein, putative, unclassified, expressed |
| R7.1 | bio5 | 9098 | 9247 | LOC_Os07g15840 | 9202349 | 9204912 | Retrotransposon, putative, centromere-specific, expressed |
| R7.1 | bio5 | 9098 | 9247 | LOC_Os07g15850 | 9206497 | 9212133 | Retrotransposon protein, putative, Ty1-copia subclass |
| R7.1 | bio5 | 9098 | 9247 | LOC_Os07g15860 | 9215115 | 9216174 | Expressed protein |
| R7.1 | bio5 | 9098 | 9247 | LOC_Os07g15870 | 9218141 | 9219332 | Expressed protein |
| R7.1 | bio5 | 9098 | 9247 | LOC_Os07g15880 | 9221388 | 9227210 | Mitochondrial prohibitin complex protein 2, putative, expressed |
| R7.1 | bio5 | 9098 | 9247 | LOC_Os07g15890 | 9230305 | 9235686 | Retrotransposon protein, putative, Ty1-copia subclass |
| R7.1 | bio5 | 9098 | 9247 | LOC_Os07g15910 | 9246587 | 9247827 | Expressed protein |
| R7.1 | bio5 | 9098 | 9247 | LOC_Os07g15920 | 9249834 | 9250283 | Expressed protein |
| R7.1 | bio5 | 9098 | 9247 | LOC_Os07g15930 | 9252392 | 9255709 | Legume lectins beta domain containing protein, expressed |
| R8.1 | bio13 | 22882 | 22883 | LOC_Os08g36300 | 22871827 | 22872433 | Expressed protein |
| R8.1 | bio13 | 22882 | 22883 | LOC_Os08g36310 | 22876101 | 22877810 | Cytochrome P450, putative, expressed |
| R8.1 | bio13 | 22882 | 22883 | LOC_Os08g36320 | 22878668 | 22882730 | Decarboxylase, putative, expressed |
| R8.1 | bio13 | 22882 | 22883 | LOC_Os08g36330 | 22888647 | 22893050 | POLE2B - Putative DNA polymerase epsilon complex subunit, expressed |
| R10.1 | bio14 | 16192 | 16195 | LOC_Os10g31000 | 16180798 | 16185035 | Ubiquitin-conjugating enzyme, putative, expressed |
| R10.1 | bio14 | 16192 | 16195 | LOC_Os10g31010 | 16188071 | 16188715 | Retrotransposon protein, putative, Ty1-copia subclass, expressed |
| R10.1 | bio14 | 16192 | 16195 | LOC_Os10g31020 | 16192110 | 16195442 | Retrotransposon protein, putative, Ty1-copia subclass, expressed |
| R10.1 | bio14 | 16192 | 16195 | LOC_Os10g31030 | 16201250 | 16208251 | EMB1691, putative, expressed |
| R12.1 | bio14 | 4400 | 4425 | LOC_Os12g08650 | 4390626 | 4391277 | Expressed protein |
| R12.1 | bio14 | 4400 | 4425 | LOC_Os12g08660 | 4394260 | 4396286 | Retrotransposon, putative, centromere-specific, expressed |
| R12.1 | bio14 | 4400 | 4425 | LOC_Os12g08670 | 4397152 | 4400640 | C2 domain containing protein, expressed |
| R12.1 | bio14 | 4400 | 4425 | LOC_Os12g08680 | 4402091 | 4404503 | Expressed protein |
| R12.1 | bio14 | 4400 | 4425 | LOC_Os12g08700 | 4412911 | 4415903 | Expressed protein |
